# Delay of Feed Post-Hatch Causes Changes in Expression of Immune-Related Genes and Their Correlation with Components of Gut Microbiota, but Does Not Affect Protein Expression

**DOI:** 10.3390/ani12101316

**Published:** 2022-05-21

**Authors:** Katarzyna B. Miska, Stanislaw Kahl, Lori L. Schreier, Beverly Russell, Kouassi R. Kpodo, Monika Proszkowiec-Weglarz

**Affiliations:** Animal Biosciences and Biotechnology Laboratory, Agricultural Research Service, United States Department of Agriculture, Beltsville, MD 20705, USA; staskahl@gmail.com (S.K.); lori.schreier@usda.gov (L.L.S.); russellbev@yahoo.com (B.R.); kouassi.kpodo@usda.gov (K.R.K.); monika.weglarz@usda.gov (M.P.-W.)

**Keywords:** delayed feeding, interleukins, avian defensins, gene expression, microbiota, chicken

## Abstract

**Simple Summary:**

Newly hatched chicks do not have access to feed until between 48 and 72 h post-hatch based on standard practices in the poultry industry. How these practices affect the chicken’s immune system in not well understood. In this study, we investigated the effect of a delay in access to feed for 48 h in newly hatched chicks on the expression of various immune-related genes in the ileum and analyzed the correlation between these genes and the components of the ileal microbiota. The results suggest that several immune-related genes were affected by delayed access to feed and the age of the birds; however, these changes were transient, occurring mostly within 48 h of the return of birds to feed. In the correlation analysis between gene expression and components of the ileal microbiota, an increased number of significant correlations between immune-related genes and the genera *Clostridium*, *Enterococcus*, and the species *Clostridium perfringens* suggests a perturbation of the immune response and ileal microbiota in response to lack of feed immediately post-hatch. These results point out the complexity of the interplay between microbiota and the immune response and will help further explain the negative effects of delay in access to feed on production parameters in chickens.

**Abstract:**

Because the delay of feed post-hatch (PH) has been associated with negative growth parameters, the aim of the current study was to determine the effect of delayed access to feed in broiler chicks on the expression of immune-related genes and select proteins. In addition, an analysis of the correlation between gene expression and components of the gut microbiota was carried out. Ross 708 eggs were incubated and hatched, and hatchlings were divided into FED and NONFED groups. The NONFED birds did not have access to feed until 48 h PH, while FED birds were given feed immediately PH. The ileum from both groups (n = 6 per group) was sampled at embryonic day 19 (e19) and day 0 (wet chicks), and 4, 24, 48, 72, 96, 144, 192, 240, 288, and 336 h PH. Quantitative PCR (qPCR) was carried out to measure the expression of avian interleukin (*IL)-1β, IL-4*, *IL-6*, *IL-8*, *IL-18*, transforming growth factor (*TGF-β*), toll-like receptor (TLR)2, TLR4, interferon (*IFN*)*-β*, *IFN-γ*, and avian β-defensins (*AvBD*) I, *2*, *3*, *5*, *6*, *7*, *8*, *9*, and *10*. Protein expression of IL-10, IL-1β, IL-8, and IL-18 were measured using ELISAs. A correlation analysis was carried out to determine whether any significant association existed between immune gene expression and components of the ileal luminal and mucosal microbiota. Expression of several immune-related genes (*TGF-β*, *TLR4*, *IFN-γ*, *IL-1β*, *IL-4*, *IL-6*, and *AvBDs 8* and *9*) were significantly affected by the interaction between feed status and age. The effects were transient and occurred between 48 and 96 h PH. The rest of the genes and four proteins were significantly affected by age, with a decrease in expression noted over time. Correlation analysis indicated that stronger correlations exist among gene expression and microbiota in NONFED birds. The data presented here indicates that delay in feed PH can affect genes encoding components of the immune system. Additionally, the correlation analysis between immune gene expression and microbiota components indicates that a delay in feed has a significant effect on the interaction between the immune system and the microbiota.

## 1. Introduction

Because birds lay eggs and do not give birth to live young, their hatchlings rely on the internalized contents of the yolk as a source of nutrients and water until the first meal is obtained. In broiler chickens, this adaptation for survival has been utilized by the poultry industry since hatchlings can survive without food or water until the contents of the yolk sac are depleted several days post-hatch [1,2]. Broiler chicken hatchlings do not have access to feed or water until they have been placed in the poultry house, which can take up to 48 h following hatch [3]. Hatchlings are usually not removed from the hatcher until most chicks have hatched, and are then sorted, vaccinated, and transported from the hatchery to grow-out facilities. Previous studies have noted that this delay in access to feed can have detrimental effects on production parameters of the growing birds, such as decrease in body weight gain, increased feed conversion ratio (FCR), and mortality [4,5,6]. Additionally, delay in access to feed can affect the development of the intestinal tract and other organs, resulting in depression of intestinal function [5,7,8,9]. Delay in access to feed for hatchlings has also been associated with changes in immunological function [10,11], therefore, in the current study, the effect of feed delay on the expression of genes associated with immune function was investigated. Additionally, a correlation analysis was performed to determine whether gene expression was related to components of the microbiota.

It has been shown, particularly in mammalian vertebrates, that nutritional status, microbiota, and immune response are tightly interwoven and dependent on one another [12,13]. Because the gastrointestinal tract is exposed to different types of nutrients and houses a diverse microbiota that can vary depending on feed, stress, and housing conditions (to name a few factors), it also houses a vast number of immune cells [14]. Kogut [15] described the environment in the gastrointestinal tract as highly antigenic. In a normally functioning gastrointestinal tract, homeostasis is in place and the immune system is tolerant of the resident microbiota while responding to pathogens. However, stress can lead to dysbiosis, where the mucosal immune response is activated and components of the microbiota undergo changes leading to inflammatory responses in the gut [16], which can decrease weight gain since mounting an immune response is bioenergetically costly [17]. In order to investigate the effects of delay in feed delivery post-hatch in broiler chickens on their immune status, a panel of genes was selected that encode proteins, which are part of the innate as well as adaptive and/or immune responses and those representing the Th1 (proinflammatory) or Th2 (anti-inflammatory) functions. These genes included avian interleukins 1β (IL-1β), 4 (IL-4), 6 (IL-6), 8 (IL-8), and 18 (IL-18), as well as transforming growth factor β (TGF-β), interferon β (IFN-β), and interferon γ (IFN-γ), which are primarily representative of the adaptive immune response.

Because there is a limited sequence identity between mammalian and chicken interferons, the identification and nomenclature of the aforementioned genes were based on activity and genomic organization [18]. IFN-β was first identified as a serologically distinct protein (from IFN-α), which was encoded by a single gene that had anti-viral activity [19]. IFN-γ is one of the classical cytokines, which functions in the induction of Th1 responses and was first isolated from a T cell line in chickens [20]. In chickens, IFN-γ is associated with oxidative burst and nitric oxide (NO) synthesis, especially when exposed to infectious agents [21]. Interferons are generally recognized as having pro-inflammatory actions and are upregulated in response to bacterial and viral pathogens [21,22].

Chicken IL-1β was initially isolated from a macrophage cell line upon being stimulated with lipopolysaccharide (LPS) [23]. IL-1β functions as a pro-inflammatory cytokine, secreted primarily by macrophages and monocytes, and activates and enhances the production of other cytokines and chemokines [24]. Chicken IL-4 was first identified and sequenced in an effort to sequence a gene cluster containing several genes involved in the Th2 response [25]. Recently, the function of IL-4 in chickens has been further elucidated, demonstrating that it can inhibit NO production by LPS stimulated macrophages/monocytes, further implicating its function as a Th2 cytokine [26]. Interleukin 10 is another cytokine that is associated with Th2 responses and is responsible for the inhibition of pro-inflammatory cytokines and NO production [27]. Chicken IL-10 was initially described by Rothwell et al. [28] and showed to be differentially expressed in chickens with different susceptibilities to intracellular parasites, underlining its importance in the Th1/Th2 paradigm. Chicken IL-6 was first isolated using subtractive hybridization methods from spleen extracts [29]. IL-6 is a multifunctional cytokine, which is involved in inflammatory responses and has been shown to have activity in *Eimeria*-infected birds [30]. IL-8 is a member of the CXC family of chemokines and serves as a chemoattractant for leukocytes, the activation of which leads to pro-inflammatory responses, such as oxidative burst and the enhancement of cell killing [31]. IL-8 is upregulated during infections and in the chicken it was first isolated from fibroblasts, where its expression was highly upregulated after transformation with Rous sarcoma virus [32]. IL-18 was initially isolated as a molecule which can induce IFN-γ and is, therefore, important in Th1 responses [33]. In chickens, IL-18 was identified and cloned [34] and was also found to be capable of inducing IFN-γ in spleen cells [35].

The TGF-β family of proteins is multifunctional and is considered to be “anti-inflammatory” (Th2) in function and play a role in achieving immune tolerance to infections, such as those caused by *Salmonella* [36]. In addition to immunological functions, TGF-β plays a role in development and growth, as well as apoptosis [37]. Other important molecules involved in innate immune responses are beta defensins. Beta defensins have a broad-spectrum antimicrobial activity [38]. The chicken genome encodes 14 AvBDs, which are cationic and are less than 100 amino acids in length [38]. Although the activity of AvBDs is focused primarily on the innate immune response, they can also enhance adaptive responses by attracting immune cells such as T lymphocytes to sites of inflammation [39]. Zhang and Wong [40] reported that AvBDs are expressed in the yolk sac, indicating that the innate immunity of newly hatched chicks is functional at hatch.

The purpose of the current study was to determine the effect of a delay in feeding newly hatched chicks on the expression of immune-related genes (and limited number of proteins) in order to examine whether a delay in feeding following hatch can have effects on the immune status of the birds. Additionally, we have examined the correlation between gene expression and the presence of various components of the microbiota at the family, genus, and species levels.

## 2. Materials and Methods

### 2.1. Animals and Husbandry Protocols

All animal husbandry methods described were approved by the Animal Care and Use Committee of the Beltsville Agricultural Research Center (BARC), in Beltsville MD. The experimental protocol used has been previously described in detail by Proszkowiec-Weglarz et al. [6,41]. Briefly, 250 fertile Ross 708 broiler eggs were obtained from a Perdue hatchery (Hurlock, MD). Eggs were incubated under standard conditions (37.5 °C and 60% humidity). All the birds used for the study were hatched between 486 and 496 h of incubation, and were then removed in 3 batches and randomly placed in battery cages such that each cage contained birds from each hatching batch. After placement, 14–15 chicks were housed in heated battery brooders. Hatchlings were divided into two treatments (n = 6 batteries per treatment). One group received feed immediately upon placement (FED), and the second did not receive feed for 48 h (NONFED). Animals in both treatment groups received water *ad libitum*. This study used “straight run” birds, and the sex of the birds was determined during sampling. All birds were fed a commercial type of corn soybean meal diet (23.7% crude protein), meeting or exceeding the National Research Council (NRC) recommendations [42].

### 2.2. Bird Sampling

Six birds were sampled at hatch from each battery (0 h, wet chicks, within 30 min from hatch), and 4, 24, 48, 72, 96, 144, 192, 240, 288, and 336 h post-hatch (PH). Additionally, embryos were sampled at embryonic day 19 (e19; n = 6, due to the small size of the embryos, tissues from two embryos were pooled together). At each sampling time, chicks and feed were weighed to determine body weight gain and feed intake for each pen and the results of the growth performance are described in detail in Proszkowiec-Weglarz et al. [6]. Beginning at 24 h PH, one chick per pen was selected at random and sacrificed by cervical dislocation. For RNA and protein isolation, 1 cm of the distal part of the ileum (approximately 10 cm anterior to the ileocecal junction) was collected, cleaned of digesta by gently pressing the tissue, and snap-frozen in liquid nitrogen. To determine luminal and mucosal bacterial populations, ileal digesta and epithelial scrapings were collected, respectively, from the distal part of the ileum (from Meckel’s diverticulum to ileocecal junction).

### 2.3. RNA Isolation and Reverse Transcription-Quantitative PCR

The RNA isolation and cDNA synthesis of the samples were described in detail by Proszkowiec-Weglarz et al. [6,41]. Briefly, total RNA was extracted using the RNeasy Mini QIAcube kit using the QIAcube instrument (Qiagen, Valencia, CA, USA), following the manufacturer’s protocol. RNA was quantified using NanoDrop One (Thermo Fisher Scientific Inc., Waltham, MA, USA). The quality of total RNA was evaluated using the 2100 Bioanalyzer (Agilent Technologies, Santa Clara, CA, USA). Two-step reverse transcription-quantitative PCR (RT-qPCR) was carried out to measure the expression of immune-related genes in the RNA from the jejunum and ileum. RT reactions (20 μL) consisted of 0.5 μg of RNA, 50 units Superscript IV reverse transcriptase (Invitrogen, Carlsbad, CA, USA), 40 units of an RNase inhibitor (Invitrogen), 0.5 mM dNTPs, and 2.5 μM anchored oligo dT primers (Milipore Sigma, St. Louis, MO; 5′CGGAATTCTTTTTTTTTTTTTTTTTTTTV-3′). A pool of all RNA (0.5 μg) from all treatment groups was used as a negative control for genomic DNA contamination and was processed in the same manner as the other samples but did not contain Superscipt IV. The RT reactions were diluted to 200 μL before being used in PCR. PCR was performed in 15 μL reactions containing 2 μL of cDNA, 400 nM of each (Forward and Reverse) gene-specific primer, SsoAdvanced™ Universal SYBR^®^ Green Supermix (Bio-Rad, Hercules, CA, USA) and was carried out in the CFX96TM Touch System (Bio-Rad). Thermal cycling parameters were as follows: 1 cycle at 95 °C for 5 min, followed by 40 cycles of 95 °C for 15 s, 60 °C for 30 s, and 72 °C for 30 s.

Dissociation curve analysis and gel electrophoresis were employed to ensure that a single PCR amplicon of the correct size was amplified. Except for AvBD4, β actin, ubiquitin, and GAPDH, all primer sequences were designed using Primer3Plus software [43] and are listed in Table 1. Avian defensin 4 (AvBD4) primers were previously published by Butler et al. [44], while primers for three housekeeping genes, β actin, ubiquitin, and GAPDH, were previously described by Proszkowiec-Weglarz [6]. Gene expression data were normalized to the geometric mean [45] of the three housekeeping genes and transformed using the equation 2^−Ct^, where Ct represents the fractional cycle number when the amount of amplified product reached a fixed threshold of fluorescence [46] The data were analyzed and are presented as fold changes relative to the values measured in e19 (–48 h) group.

### 2.4. Protein Extraction and ELISA

Ileal samples were homogenized using ice-cold T-Per^®^ tissue protein extraction reagent (Thermo Fischer Scientific, Inc., Waltham, MA, USA) containing 1 mM phenylmethylsulphonyl fluoride (Thermo Fisher Scientific, Inc.) and Halt™ protease and phosphatase inhibitor cocktail (Thermo Fisher Scientific, Inc.), and the homogenate was centrifuged at 10,000× *g* for 10 min. Protein concentration in collected supernatant was quantified using the Coomassie Plus (Bradford) assay kit (Thermo Fisher Scientific, Inc.). Protein expression levels of IL-1β, IL-8, IL-10, and IL-18 in ileal tissue homogenates were determined using a commercially available chicken ELISA kit (LifeSpan BioSciences, Inc., Seattle, WA, USA) according to the manufacturer’s instructions. Before the assay, original ileum homogenates were diluted with the kit’s sample diluent 1:26 for IL-8 and IL-10, 1:121 for IL-1β, and 1:961 for IL-18. The optical density was determined using plate reader (SpectraMax M2, Molecular Devices, San Jose, CA, USA) set to 450 nm. Results were calculated from standard curve using SpectraMax M2 software.

### 2.5. DNA Isolation, Library Preparation, and Microbiota Analysis

The DNA isolation, library preparation, and microbiota analysis have been previously described by Proszkowiec-Weglarz et al. [41]. Briefly, DNA was extracted from each sample using a DNeasy PowerSoil kit (Qiagen, Valencia, CA, USA) and a QIAcube instrument (Qiagen) as per the manufacturer’s protocol. DNA concentration and quality were assessed by NanoDrop (ThermoFisher Scientific, Inc., Waltham, MA, USA) and TapeStation System (Agilent Technologies). The 16S rRNA gene amplicon libraries were generated using the Illumina chemistry and workflow (Illumina, Inc., San Diego, CA, USA), and PCR primers targeted the V3–V4 variable region of the 16S gene. The pooled DNA library was diluted to a final concentration of 4 pM and mixed with PhiX (Illumina, Inc., San Diego, CA, USA, 4 nmol) control (20% *v*/*v*). The pair-end 2 × 300 bp sequencing was performed using the Illumina MiSeq platform and a MiSeq Reagent Kit v3 (Illumina, Inc, San Diego, CA, USA). The relative abundance of bacterial families, genera, and species used for correlation analysis has been determined as described in Proszkowiec-Weglarz et al. [47]. Unclassified bacteria were determined as unclassified bacterial reads at the specific taxonomic level. Other bacteria were determined as differences between total bacterial reads for a sample and named bacterial taxa in that sample. The 16S rRNA gene sequences used in this study were previously deposited in the NCBI Sequence Read Archive (SRA) database (SRA accession # PRJNA779402).

### 2.6. Statistical Analysis

Gene expression data were analyzed by two-way ANOVA using the general linear models (SAS). Age (represents h post-hatch), treatment (FED and NONFED), and their interaction were set as the fixed effects. Main effects were not analyzed separately if the interaction between them was significant. Significance was set at *p* < 0.05. To evaluate the relationship between the expression of selected immune-related genes and microbiota, a Pearson correlation coefficient was calculated between gene expression and the relative abundance of bacterial population in the ileum using GraphPad Prism version 9.1.0 for Windows (GraphPad Software, San Diego, CA, USA). All calculations were performed separately for FED and NONFED birds with the data pooled across all time points (24 h to 336 h PH). All reported *p*-values for correlation coefficients were adjusted to control the experimentwise error rate with a false discovery rate (FDR) approach using a two-stage linear step-up procedure of GraphPad Prism. Heat maps representing a significant relationship (r, *p* < 0.05) between gene expression and the respective bacterial relative abundance were also constructed with GraphPad Prism software.

## 3. Results

### 3.1. Gene Expression

#### 3.1.1. Cytokines and Toll-Like Receptors

**Figure 1:**
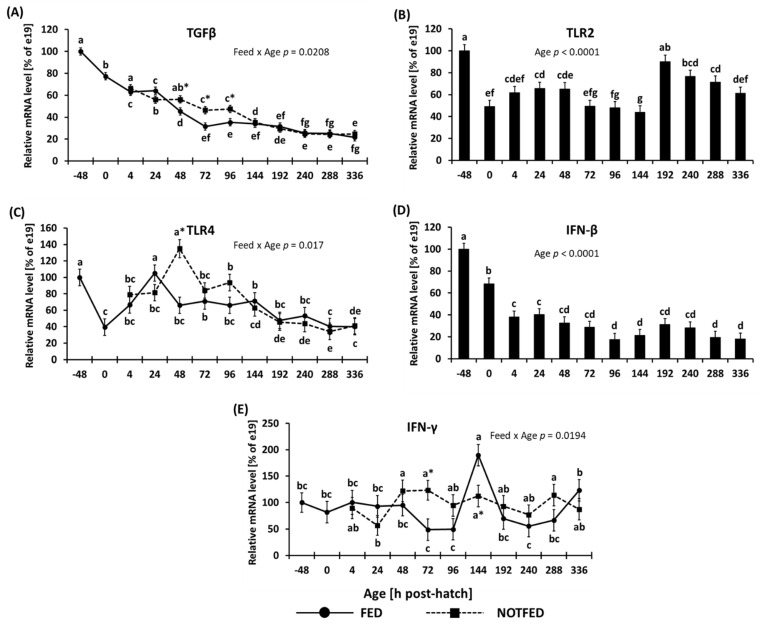
Effect of delay in access to feed on mRNA levels of cytokines in the ileum: (**A**) *TGF-β*, (**B**) *TLR2*, (**C**) *TLR4*, (**D**) *IFN-β*, and (**E**) *IFN-γ*. Figure 1A,C,E show two-way ANOVA results where significant (*p* < 0.05) interaction between treatment and age influenced gene expression. Figure 1B,D shows one-way ANOVA results where age had a significant (*p* < 0.05) effect on gene expression. Gene expression was calculated to be relative to expression at e19 (−48 h), where expression at e19 was set to 100%, and subsequent timepoints are presented as % of the e19 (−48 h) values. Each value represents a mean ± SE of 6 birds. Different letters denote statistically significant (*p* < 0.05) values within a treatment. An asterisk (*) denotes significant (*p* < 0.05) difference between FED (fed immediately after hatch) and NONFED (48 h delayed access to feed) treatments.

The expression of *TGF-β* is shown in Figure 1A. Following hatch, a decrease in expression *TGF-β* was detected in both FED and NONFED birds. The expression in FED birds decreased more rapidly than seen in NONFED birds, after which the expression levels stabilized. Due to a more rapid decline in expression in FED birds, between 48 and 96 h PH the NONFED birds had significantly increased expression of *TGF-β* compared to FED birds (Figure 1A). No differences in *TGF-β* expression were observed from 144 h to 336 h PH between the treatment groups (Figure 1A).

Expression of *TLR2* is shown in Figure 1B. There was no interaction between the main effects; however, a significant effect of age (*p* < 0.0001) was found. A decrease in expression was observed between e19 embryos (−48 h) and hatchlings. The levels of expression remained steady until 144 h PH, but at 192 h PH the expression of *TLR2* was more than double that of 144 h. Between 192 h and 336 h the expression of *TLR2* once again decreased (Figure 1B).

The expression of *TLR4* is shown in Figure 1C. The two-way ANOVA indicated a significant (*p* = 0.017) interaction between age and feed status. In NONFED birds, the expression of *TLR4* was significantly greater at 48 h than in FED birds. However, by 72 h PH the expression of *TLR4* was similar between FED and NONFED birds. A decrease in expression of TLR4 was observed at day 0 compared up to 48 h (Figure 1C).

The expression of *IFN-β* is shown in Figure 1D. Interaction between the main effects was not significant, however, a significant effect of age (*p* < 0.0001) was found. *IFN-β* gene expression decreased from −48 h to 0, and 4 h PH, after which the expression remained steady from 4 h to 336 h PH (Figure 1D).

The expression of *IFN-γ* is shown in Figure 1E. The two-way ANOVA indicated a significant interaction between age and feed status (*p* = 0.0194). The expression of *IFN-γ* was fairly steady throughout the course of the study; however, at 72 h PH NONFED birds had significantly higher expression compared to FED birds, but at 144 h PH, NONFED birds had significantly lower expression of *IFN-γ* due to a large upward spike in expression in FED birds. Following the spike, the expression of *IFN-γ* was reduced to levels observed at earlier timepoints (Figure 1E).

**Figure 2:**
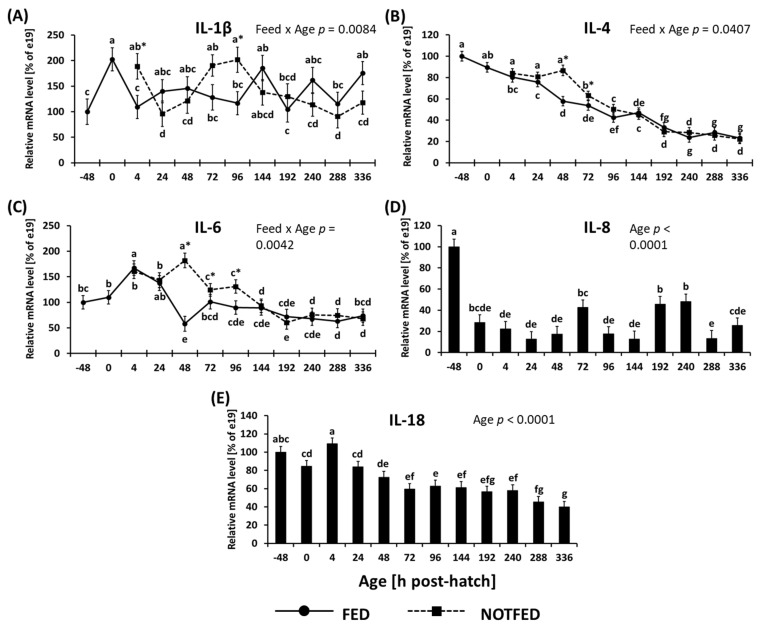
Effect of delay in access to feed on mRNA levels of cytokines in the ileum: (**A**) *IL-1β*, (**B**) *IL-4*, (**C**) *IL-6*, (**D**) *IL-8*, and (**E**) *IL-18*. Figure 1A–C shows two-way ANOVA results where significant (*p* < 0.05) interaction between treatment and age influenced gene expression. Figure 1D,E show one-way ANOVA results where age had a significant (*p* < 0.05) effect on gene expression. Gene expression was calculated to be relative to expression at e19 (−48 h), where expression at e19 was set to 100%, and subsequent timepoints are presented as % of the e19 (−48 h) values. Each value represents a mean ± SE of 6 birds. Different letters denote statistically significant (*p* < 0.05) values within a treatment. An asterisk (*) denotes significant (*p* < 0.05) difference between FED (fed immediately after hatch) and NONFED (48 h delayed access to feed) treatments.

The expression of *IL-1β* is shown in Figure 2A. Two-way ANOVA indicated that there was a significant interaction between age and treatment (*p* = 0.0084) where *IL-1β* expression in NONFED birds was significantly higher at 4 and 96 h PH. In FED birds, the expression increased at day of hatch before dropping back down to levels observed at −48 h to 4 h PH. In NONFED birds, expression of *IL-1β* decreased at 4 h PH, then underwent an increase which peaked at 96 h, followed by another decrease in expression, and steady expression levels for the remainder of the study (Figure 2A).

The expression of *IL-4* is shown in Figure 2B. Two-way ANOVA indicated that there was a significant interaction between age and treatments (*p* = 0.0407) where *IL-4* expression in NONFED birds was significantly higher at 48 and 72 h. The expression of *IL-4* in FED birds began to decrease beginning at 0 h PH and continued to decrease gradually until 240 h, while in NONFED birds the decrease in expression did not begin until 72 h PH (Figure 2B).

The expression of *IL-6* is shown in Figure 2C. Two-way ANOVA indicated that there was a significant interaction between age and treatment (*p* = 0.0042) where *IL-6* expression in NONFED birds was significantly higher between 48 and 96 h. The expression of IL-6 in FED birds initially increased at 4 h PH followed by a decrease at 48 h and a small increase at 72 h. The expression of *IL-6* remained stable for remainder of the study. Meanwhile, in NONFED birds an increase in expression was observed at 48 h followed by a decrease in expression and a stabilization for the remainder of the study (Figure 2C).

Expression of *IL-8* is shown in Figure 2D. There was an absence of significant interaction between feed status and age; however, a significant age effect was observed (*p* < 0.0001). The expression of *IL-8* decreased between −48 and 48 h by more than 3 times of the level observed at −48 h. For the remainder of the study, the expression of *IL-8* stayed at fairly constant levels never exceeding half the level observed at −48 h (Figure 2D).

The expression of *IL-18* is shown in Figure 2E. There was no significant interaction between feed status and age; however, a significant age effect was observed (*p* < 0.0001). Once again, a decrease in expression occurred over time; however, the decrease was gradual and effectively began at 72 h and was maintained until the end of the study (Figure 2E).

#### 3.1.2. Avian Defensins

**Figure 3:**
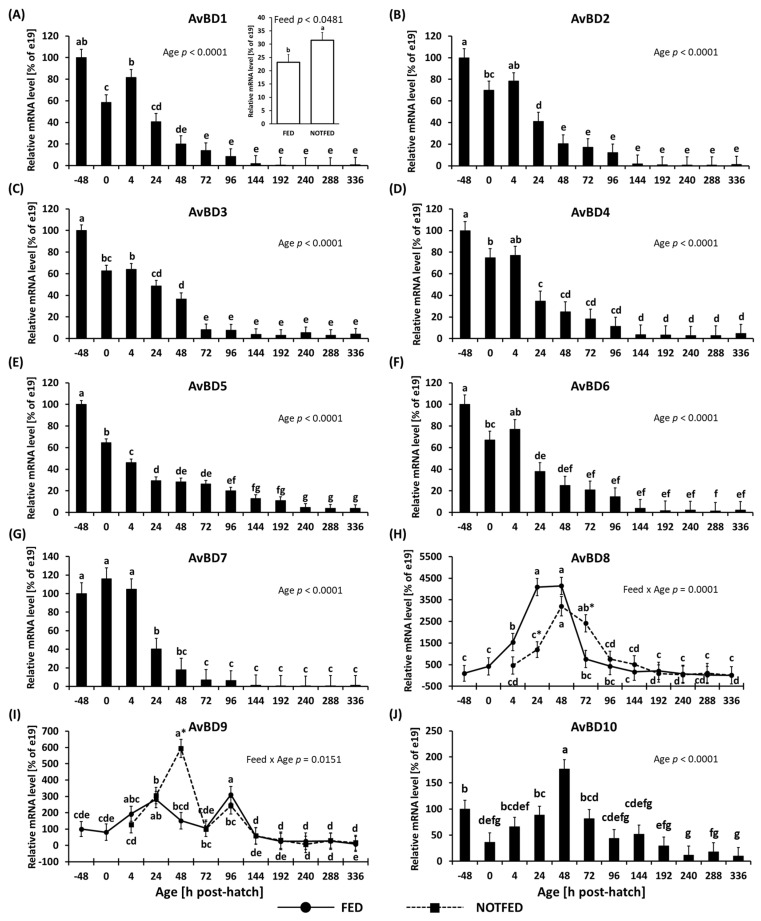
Effect of delay in access to feed on mRNA levels of avian defensins in the ileum: (**A**) *AvBD1*, (**B**) *AvBD2*, (**C**) *AvBD3*, (**D**) *AvBD4*, (**E**) *AvBD5*, (**F**) *AvBD6*, (**G**) *AvBD7*, (**H**) *AvBD8*, (**I**) *AvBD9*, and (**J**) *AvBD10*. Figure 3H,I shows two-way ANOVA results where significant (*p* < 0.05) interaction between treatment and age influenced gene expression. The inset of 3A shows significant (*p* < 0.05) differences in gene expression between FED and NONFED birds. Figure 3A–G,J show one-way ANOVA results where age had a significant (*p* < 0.05) effect on gene expression. Gene expression was calculated to be relative to expression at e19 (−48 h), where expression at e19 was set to 100%, and subsequent timepoints are presented as % of the e19 (−48 h) values. Each value represents a mean ± SE of 6 birds. Different letters denote statistically significant (*p* < 0.05) values within a treatment. An asterisk (*) denotes significant (*p* < 0.05) difference between FED (fed immediately after hatch) and NONFED (48 h delayed access to feed) treatments.

The expression of avian defensin genes 1–10 is shown in Figure 3. Expression of *AvBDs* 1–7 and *AvBD10* showed a significant effect of age (Figure 3A–G,J). The mRNA of *AvBDs* 1–7 decreased in expression beginning at 0 h (with the exception of *AvBdD7* whose expression remained steady through 4 h).

By 72 h their expression reached steady levels. The decrease in expression of these genes was large and by 336 h it decreased by more than 99% compared to values observed at −48 h. The expression of *AvBD1* was significantly (*p* = 0.0481) higher in NONFED birds (Figure 3A inset). Expression of *AvBD8* and *9* (Figure 3H,I) showed a significant interaction between age and feed status (*p* = 0.0001 and *p* = 0.0151, respectively). The expression of *AvBD8* was lower at 24 h, but greater at 72 h PH in NONFED compared to FED birds. The FED birds showed an increase in expression beginning at 4 h, reaching its peak at 48 h, followed by decrease in expression. NONFED birds followed a similar expression pattern as FED birds. By 192 h, the expression of *AvBD8* in both FED and NONFED birds remained steady (Figure 3H). The expression of *AvBD9* was significantly higher at 48 h in NONFED birds (Figure 3I). This peak in expression was followed by a large drop at 72 h, after which both FED and NONFED birds followed a similar expression pattern, with steady expression continuing from 144 h until the end of the study at 336 h. The expression of *AvBD10* is shown in Figure 3J. The expressions of *AvBD10* showed a significant effect of age (*p* < 0.0001), where expression decreased from −48 h to 0 h followed by an increase in expression, which peaked at 48 h followed by a gradual decrease (Figure 3J). 

### 3.2. Protein Expression of Cytokines

**Figure 4:**
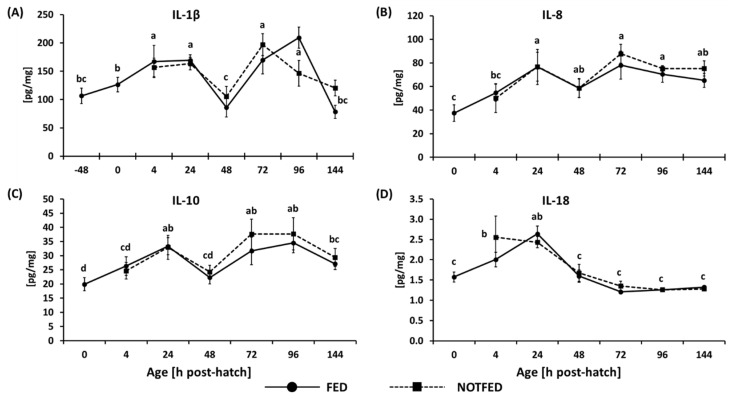
Effect of delay in access to feed on ileal protein levels for (**A**) IL-1β, (**B**) IL-8, (**C**) IL-10, and (**D**) IL-18 determined by ELISA. Each value represents mean ± SE of 6 birds. Different letters denote statistically significant (*p* < 0.05) differences between age (h post-hatch).

Currently, only limited reagents exist which recognize or cross-react with chicken proteins; however, we were able to carry out ELISAs on four cytokines, IL-1β, IL-8, IL-10, and IL-18, on samples collected between −48 h (IL-1β) or 0 h (IL-8, IL-10, and IL-18) and 144 h. The results of the assays conducted on proteins extracted from the ileum are shown in Figure 4. No treatment by age interaction or treatment effects was observed; however, significant differences were observed between timepoints.

Expression of IL-1β is shown in Figure 4A. After hatch, an increase in protein concentration was seen at 4 and 24 h, followed by a decrease at 48 h, and another increase between 72 and 96 h. At 144 h, the amount of IL-1β present in the ileum once again decreased. Expression of the IL-8 protein is shown in Figure 4B. Concentration of IL-8 in the ileum increased between hatch and 24 h after which it remained fairly constant. The expression of the IL-10 protein is shown in Figure 4C. The concentration increased from hatch to 24 h, followed by a decrease in concentration at 48 h, and another increase between 72 and 96 h. By 144 h the level of the IL-10 protein was again decreased. The expression of IL-18 is shown in Figure 4D. The amount of IL-18 present was highest at 24 h, followed by a decrease at 48 h and steady protein levels until 144 h.

### 3.3. Correlation among mRNA Expression of Immune-Related Genes and Microbiota Composition

**Figure 5:**
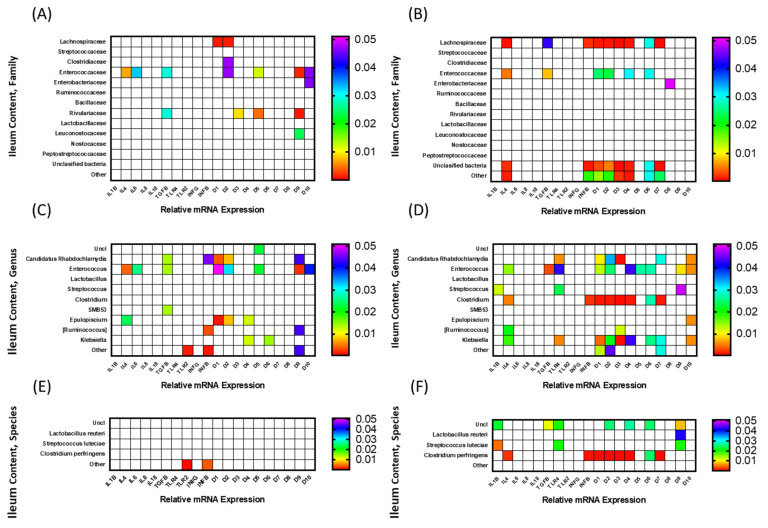
Heat map of correlations (P of Pearson’s r) between luminal microbiota and expression of immune-related genes in ileum. Comparisons (**A**) at family level in FED and (**B**) NONFED birds, (**C**) at genus level in FED and (**D**) NONFED birds, and (**E**) at species level in FED and (**F**) NONFED birds. FED birds had immediate access to feed after hatch, while NONFED birds had 48 h delay in access to feed.

**Figure 6:**
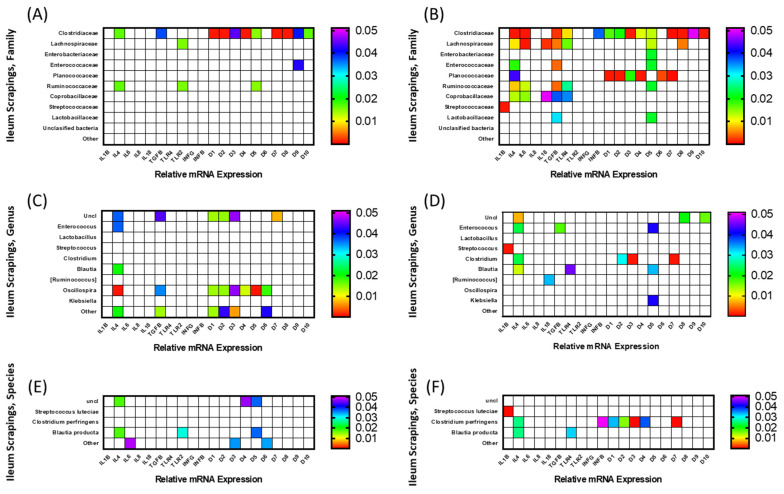
Heat map of correlations (P of Pearson’s r) between mucosal microbiota and expression of immune-related genes in ileum. Comparisons (**A**) at family level in FED and (**B**) NONFED birds, (**C**) at genus level in FED and (**D**) NONFED birds, and (**E**) at species level in FED and (**F**) NONFED birds. FED birds had immediate access to feed after hatch, while NONFED birds had 48 h delay in access to feed.

To determine whether relationships exist between mRNA expression of immune-related genes and components of the microbiota, a correlation analysis was carried out and significant (*p* < 0.05) interactions are displayed as heat maps in Figure 5 and Figure 6. The microbiota composition was determined for both ileal contents and scrapings of FED and NONFED birds. The correlation analysis of ileal content (luminal bacterial population) is shown in Figure 5, and that of ileal scrapings (mucosal bacterial population) in Figure 6. The analyses were carried out at the family, genus, and species levels.

#### 3.3.1. Ileal Luminal Microbiota

The correlation among gene expression in FED and NONFED birds and constituents of the microbiota at the family level in ileal content are shown in Figure 5A,B, respectively. In FED birds, the presence of 6 bacterial families (Lachnospiraceae, Leuconostocaceae Clostridiaceae, Enterococcaceae, Enterobacteriaceae, and Rivulariaceae) were found to be significantly correlated with the expression of 9 immune-related genes (*IL-4*, *IL-6*, *TGF-β*, and *AvBD1*, *2*, *3*, 5, *9*, and *10*). Positive correlation was found among gene expression and microbiota in all cases with the exception of Enterococcaceae. The family Enterococcaceae was negatively correlated with the expression of 7 genes (*IL-4*, *IL-6*, *TGF-β*, *AvBD2*, *5*, *9*, and *10*), followed by the family Rivulariaceae, which was positively correlated with the expression of 4 genes (*TGF-β*, and *AvBD3*, *5*, and *9*), and Lachnospiraceae, which was positively correlated with the expression of 2 genes (*AvBD1* and *2*). The remaining 2 families, Clostridiaceae and Enterobacteriaceae, were positively correlated with 1 gene (*AvBD2* and *10*, respectively).

Figure 5B shows the correlation between microbiota families and gene expression in NONFED birds. Five families (Lachnospiraceae, Enterococcaceae, Enterobacteriaceae, unclassified bacteria, and other bacteria) were correlated with the expression of 10 genes (*IL-4*, *TGF-β*, *IFN-β*, *AvBD1-4*, *6*, *7*, and *8*). All significant correlations between gene expression of the bacterial family were positive. The Lachnospiraceae was correlated with the expression of 9 genes (*IL-4*, *TGF-β*, *IFN-β*, *AvBD1-4*, *6*, and *7*). Unclassified bacteria and the other category were correlated with the expression of 8 genes (*IL-4*, *IFN-β*, *AvBD1-4*, *6*, and *7*) and Enterococc aceae was correlated with 6 genes (*IL-4*, *TGF-β*, *AvBD1*, *2*, *4*, and *6*). The Enterobacteriaceae was associated with the expression of 1 gene (*AvBD8*). In NONFED birds, the expression of all but 3 *AvBD* genes were correlated with 5 bacterial families.

The correlation among gene expression in FED and NONFED birds and constituents of the microbiota at the genus level in ileal contents are shown in Figure 5C,D, respectively. In ileal contents of FED birds (Figure 5C), members of 8 genera (Unclassified, *Candidatus rhabdochlamydia*, *Enterococcus*, *SMB53*, *Epulopiscium*, *Ruminococcus*, *Klebsiella*, and other) were significantly correlated with the expression of 12 genes (*IL-4*, *IL-6*, *TGF-β*, *TLR-2*, *IFN-β*, and *AvBD1*, *2*, *4-6*, *9*, and *10*). Only the genus *Enterococcus* was negatively correlated the expression of 8 genes (*IL-4*, *IL-6*, *TGF-β*, and *AvBD1*, *2*, 5, *9*, and *10*). *Candidatus rhabdochlamydia* was positively correlated with the expression of 5 genes (*TGF-β*, *IFN-β*, and *AvBD1*, *2*, and *9*), with the remainder (*SMB53*, *Epulopiscium*, *Ruminococcus*, *Klebsiella*, and other) being positively correlated with the expression of 1 gene (*TGF-β*), 4 genes (*IL-4*, *AvBD1*, *2*, and *4*), 2 genes (*AvBD5* and *6*), and 3 genes (*TLR-2*, *IFN-β*, and *AvBD9*), respectively. The genus *Clostridium* was not significantly correlated with the expression of any of the genes tested in this study in the ileum contents of FED birds.

Figure 5D shows the results of the correlation analysis between immune-related genes and the presence of different bacterial genera in NONFED birds. Members of 8 genera (*Candidatus rhabdochlamydia*, *Enterococcus*, *Streptococcus*, *Clostriudium*, *Epulopiscium*, *Ruminococcus*, *Klebsiella*, and other) were significantly correlated with the expression of 14 genes (*IL-1B*, *IL-4*, *TGF-β*, *TLR-4*, *IFN-β*, *AvBD1-7*, *9* and *10*). The genus *Enterococcus* was negatively correlated with the expression of 10 genes (*IL-4*, *TGF-β*, *TLR-4*, *AvBD1*, *2*, *4-6*, *9* and *10*).

Genera *Klebsiella, Clostridium,* and *Candidatus rhabdochlamydia* were positively correlated with expression of 9 genes (*IL-4*, *TLR-4*, *AvBD1-4*, *6*, *7*, and *10*), 8 genes (*IL-4*, *IFN-β*, *AvBD1-4*, *6* and *7*), and 6 genes (*TLR-4*, *AvBD1-3*, *7* and *10*), respectively. The remaining 4 genera (*Streptococcus*, *Epulopiscium*, *Ruminococcus*, and other) were positively correlated with 3 genes (*IL-1β*, *TLR-4*, and *AvBD9*), 1 gene (*AvBD10*), 2 genes (*IL-4* and *AvBD3*) and 3 genes (AvBD1, 2, and 7), respectively.

The correlation among gene expression in FED and NONFED birds and constituents of the microbiota of ileal contents at the species level are shown in Figure 5E,F, respectively. In FED birds (Figure 5E), only bacteria belonging to “other” species were positively correlated with the expression of *TLR2* and *IFN-β*. On the other hand, in NONFED birds (Figure 5F), members representing unclassified bacterial, *Lactobacillus reuteri*, *Streptococcus luteciae*, and *Clostridium perfringens* were correlated with the expression of 12 genes (*IL-1β*, *IL-4*, *TGF-β*, *TLR4*, *IFN-β*, *AvBD1-4*, *6*, *7*, and *9*). Only unclassified bacteria were negatively correlated with the expression of 7 genes (*IL-1β*, *TGF-β*, *TLR4*, *AvBD2*, *4*, *6*, and *9*). *Clostridium perfringens* was positively correlated with the expression of 8 genes (*IL-4*, *IFN-β*, *AvBD1-4*, *6*, and *7*). The remaining 2 species (*Streptococcus luteciae* and *Lactobacillus reuteri*) were positively correlated with 3 genes (*IL-1β*, *TLR4*, and *AvBD9*) and 1 gene (*AvBD9*), respectively.

#### 3.3.2. Ileal Scrapings

The correlation among gene expression in FED and NONFED birds and constituents of the microbiota at the family level in ileal scrapings are shown in Figure 6A,B, respectively. In FED birds, 4 bacterial families (Clostridiaceae, Lachnospiraceae, Enterococcaceae, and Ruminococcaceae) were found to be significantly correlated with the expression of 12 immune-related genes (IL-4, *TGF-β*, *TLR2*, *AvBD1-5*, and *AvBD7-10*). Eleven of these genes (with the exception of *TLR2*) were positively correlated with presence of Clostridiaceae. Ruminococcaceae were positively correlated with *TLR2* expression and negatively correlated with the expression of *IL-4* and *AvBD5*. Lachnospiraceae and Enterococcaceae were each correlated positively with *TLR2*, and negatively with *AvBD5*, respectively.

Figure 6B shows correlation between microbiota families and gene expression in NONFED birds. Nine families (only members of unclassified and “other” families did not show any significant correlation with gene expression) correlated with the expression of 17 genes (*IL-1β*, *IL-4*, *IL-6*, *IL-18*, *TGF-β*, *TLR-4*, *IFN-β*, and *AvBD1-10*). Only *IL-8*, *TLR2* and, *IFN-γ* were not significantly correlated with any microbial families. Lachnospiraceae, Enterococcaceae, Ruminococcaceae, and Coprobacillaceae were negatively correlated with 7 genes (*IL-4*, *IL-6*, *TGF-β*, *TLR-4*, and *AvBD1*, and *2*), 3 genes (*IL-4*, *TGF-β*, and *AvBD5*), 5 genes (*IL-4*, *IL-6*, *TGF-β*, *TLR-4*, and *AvBD5*), and 5 genes (*IL-4*, *IL-6*, *IL-18*, *TGF-β*, *TLR-4*, *IFN-β*), respectively. Clostridiaceae, Enterobacteriaceae, Planococcaceae, Streptococcaceae, and Lactobacillaceae were positively correlated with 14 genes (*IL-4*, *IL-6*, *TGF-β*, *TLR-4*, *IFN-β*, and *AvBD1-5*, and *7-10*), 1 gene (*AvBD5*), 7 genes (*IL-4*, *IL-6*, *AvBD1-4*, *6*, and *7*), 1 gene (*IL-1β*), and 2 genes (*TGF-β* and *AvBD5*), respectively.

The correlation among gene expression in FED and NONFED birds and constituents of the microbiota at the genus level in ileal scrapings are shown in Figure 6C,D, respectively. In ileal scrapings of FED birds (Figure 6C), members of 5 genera (Unclassified, *Enterococcus*, *Blautia*, *Oscillospira*, and other) were significantly correlated with the expression of 9 genes (*IL-4*, *TGF-β*, and *AvBD1-7*). *Oscillospira* showed negative correlation with the expression of 8 (*IL-4*, *TGF-β*, and *AvBD1-6*) genes, while those belonging to “other” and unclassified bacteria were positively correlated with the expression of 6 (*IL-4*, *TGF-β*, *AvBD1-3*, *6* and *7*) genes. The remaining 2 genera, *Blautia* and *Enterococcus*, were negatively correlated with the expression of *IL-4*.

Figure 6D shows the results of the correlation analysis between immune-related genes and the presence of different bacterial genera in NONFED birds. Members of 7 genera (Unclassified, *Enterococcus*, *Streptococcus*, *Clostridium*, *Blautia*, *Ruminococcus*, and *Klebsiella*) were significantly correlated with the expression of 11 genes (*IL-1β*, *IL-4*, *IL-18*, *TGF-β*, *TLR4*, *AvBD2*, *3*, *5*, *7*, *8* and *10*). The genus *Clostridium* was positively correlated with the expression of 4 genes (*IL-4*, *AvBD2*, *3* and *7*). *Enterococcus* and *Blautia* were negatively correlated with the expression of 3 genes (*IL-4*, *TGF-β*, and *AvBD5*) and 3 genes (*IL-4*, *TLR4*, and *AvBD5*), respectively, while *Ruminococcus* and *Klebsiella* were negatively correlated with only 1 gene (*IL-18* and *AvBD5*, respectively). Unclassified bacteria and *Streptococcus* were positively correlated with the expression of 3 (*IL-4*, and *AvBD8* and *10*) and 1 (*IL-1β*) genes, respectively.

The correlation among gene expression in FED and NONFED birds and constituents of the microbiota of ileal scrapings at the species level are shown in Figure 6E,F, respectively. In FED birds (Figure 6E), unclassified bacteria were positively correlated with the expression of *IL-4*, *AvBD4*, and *AvBD5*. *Blautia* was negatively correlated with *IL-4* and *AvBD5* expression, and positively correlated with *TLR2*. Members of other species were positively correlated with the expression of *IL-6*, *AvBD6* and *7*. In NONFED birds (Figure 6F) *Streptococcus luteciae* was positively correlated with the expression of *IL-1β*, while members of the *Blautia* species were negatively correlated with the expression of *IL-4* and *TLR4*. *Clostridium perfringens* was positively correlated with the expression of *IL-4*, *IFN-β*, and *AvBD1-4* and *7*.

## 4. Discussion

The purpose of the study was to investigate the effects of delay in feeding PH on components of the innate as well as the adaptive immune response, and to determine whether gene expression data correlates with specific members of microbiota. The expression of 20 genes was measured between −48 h before hatch and 336 h PH, and 8 of them (*TGF- β, TLR4*, *IFN-γ*, *IL-1β*, *IL-4*, *IL-6*, *AvBD8*, and *AvBD9*) were shown to have a significant interaction between age and treatment, one (AvBD1) was affected by feed status, and twelve (*TLR2*, *IFN-β*, *IL-8*, *IL-18*, *AvBD1-7*, and *AvBD10*) were significantly affected by bird age. Therefore, the expression of all genes measured for this study were influenced by at least one of the main effects. Significant effects of interactions between age and feed status were seen in genes that are considered parts of the adaptive (i.e., IFN-γ) or innate immune response (AvBDs 8 and 9). Similarly, components of the Th1 (IL-1β) as well as Th2 (TGF-β) immune responses showed significant interactions between age and feed status. Therefore, it can be concluded that gene expression was affected almost 50% of the time by feed status and age, although there was no clear delineation in the functional category of the gene. Interestingly, in all 8 cases where a significant age by feed status interaction was observed, gene expression was higher in NONFED birds and occurred between 4 and 96 h PH. Only the expression of one gene, *IL-1β*, was significantly higher at 4 h PH, with the bulk of the rest of the genes being expressed higher in NONFED birds between 48 and 96 h PH. The immune system PH in chickens is influenced by early feeding and takes more time to become fully functional [10,48,49]. However, elements of the innate immune system such as TLRs are present and can provide the hatchings with some protection against pathogens [50]. In the current study, *TLR4* was upregulated between 48 and 96 h PH in NONFED birds. It is possible that NONFED birds were exposed to pathogens when they had no access to feed, and the *TLR4* gene was upregulated in response. Consequently, we observed an increase in the expression of immune-related genes (*IL-1β*, *IL-6*) because *TLR4* is involved in pathways that produce these pro-inflammatory cytokines [51]. Another pro-inflammatory cytokine, IFN-γ, was also upregulated. IFN-γ is produced by different cell types, including NK, T, and B cells, and it is involved in inflammatory responses and the elimination of pathogens [52,53]. The increase in *IFN-γ* gene expression may be due to exposure to pathogens when NONFED birds did not have access to feed as previously hypothesized. However, because birds in the current study were not challenged and no disease was observed, this hypothesis should be verified in future research. Alternatively, the lack of access to feed PH for 48 h may have delayed the distribution of T and B cells in the gut-associated lymphoid tissue (GALT) [48]. The return to feed, which likely contains antigens, may have accelerated the GALT development [48] and, thus, increased the expression of IFN-γ.

In addition, to pro-inflammatory cytokines, anti-inflammatory cytokines IL-4 and TGF-β were also upregulated. These cytokines are important for maintaining intestinal immune homeostasis [54]. Reasons of their increase following the 48-h delay in access to feed is unclear but may be related to their anti-inflammatory role in balancing the immune system in the gut. Additionally, TGF-β is involved not only in the mucosal immune system but also in wound healing and the proliferation of intestinal epithelial cells [54]. Therefore, it is possible that the upregulation of TGF-β in the current study are related to intestinal mucosal healing and repair because the delay in access to feed for 48 h PH damaged or reduced the development of the intestinal mucosa [41]. In addition, delay in access to feed delayed the expression of mucin 2 gene expression [41]. Together, these results suggest that the lack of feeding PH may disturb the immune system development and maturation in chickens.

Several studies have been published which implicate delay in feed post-hatch as a negative factor in the development and function of the immune system [10,11]; however, not many studies have measured expression of immune-related genes and proteins. Simon et al. [55] reported data from a study in which layer and broiler chicks were not fed for 72 h PH and their performance parameters as well the expression of select genes were followed for 42 days PH. The authors reported that there was no effect of delaying the feed PH in either broilers or layers on the expression of cytokines in the ileum. This study included several Th1 (IL-12p40, IL-1β, IFN-γ), Th2 (IL-10 and TGF-β), as well as several immunoglobulin genes (IgM, IgY, and IgA). In the current study, the expression of IL-1β, IFN-γ, and TGF- β were affected by the interaction between age and feed. The discrepancy in the results could be due to sampling intervals, differences in bird genotypes, diets, or other environmental differences. In the current study, samples were collected at hatch and on a daily basis (or more frequently) during the first half of the study, versus fewer samplings over a longer time reported in the previous study [55]. Simon et al. [55] also reported that broiler chickens showed a decrease in cytokine gene expression between 2 and 3 weeks PH, while in the current study, the decrease in expression began earlier. Bar Shira et al. [48] described the expression patterns of interleukin 2 (IL-2) in the duodenum and colon of Ross broilers without access to feed and water for 72 h PH. It was found that the expression of IL-2 was inhibited by a delay in feed in both sections of the gut; however, the inhibition was more profound in the hindgut. By day 12 PH the expression of IL-2 returned to levels observed in normally fed chickens. Lamot et al. [56] reported that serum concentrations of IFN-γ were higher in direct-fed chicks compared to those which were delayed access to feed for 48 h. In the context of decreased IL-2 expression, Lamot et al. [56] speculated that T cells in the gut are inhibited, which results in decreased IFN-γ production. In the current study, the effect of an interaction between age and treatment was associated with IFN-γ gene expression, however, by two weeks PH no differences were found. The data documenting the expression of cytokines in the gut of feed-delayed chickens is very limited, however, in all cases, the effects of feed-delay were transient indicating that the immune function of feed-delayed birds was able to return to normal function. Interestingly, the four cytokines (IL-1β, IL-8, IL-10, IL-18) tested during this study at the protein level were not affected by a delay in feed, underlining the importance of testing protein as well as gene expression since often the two are not parallel. As more chicken specific reagents are produced, these comparisons can be more easily performed.

Although not all the genes and none of the proteins investigated in this study were affected by a delay in feed, all were affected by age, particularly the genes of avian beta defensins, which decreased in expression over the two-week sampling period. In a recent study, Song et al. [57] investigated the effects of age (up to 34 days PH) on many immune-related responses and postulated that components of the immune response can be described as being regulated in either “down-up”, “up-down”, and “up-up” fashion. They concluded that between days 6 and 13 PH the immune system is not fully developed due to the observed decrease in peripheral blood cytokine levels and gene expression of cytokines in the intestinal mucosa. However, the results in the current study (where overlap occurred) showed several discrepancies. The gene expression of *IFN-γ* and *IL-1β* stayed fairly constant after hatch (as opposed to increased), *TGF-β* decreased after hatch (as opposed to steady expression). Song et al. [57] hypothesized that when the chick embryo develops, Th2 (anti-inflammatory) responses predominate, but following hatch, as the newly hatched chick is exposed to novel environment and antigens, a Th1 response (pro-inflammatory) develops, and a balance between Th1/Th2 responses is achieved by approximately 30 days PH. This is an intriguing hypothesis; however, there is great disparity in the published data, especially at the gene expression level. It is likely that bird genotype, environmental conditions, diet, and organs sampled create variability that is seen between studies. In the four cytokines, which were investigated at the protein level (IL-1β, IL-10, IL-8, and IL-18), two increased (IL-10 and IL-8), and two did not differ (IL-1β and IL-18) between hatch and 2 weeks PH. Interestingly, the IL-1β gene and protein expression had similar expression, but the expression of the IL-18 and IL-8 gene and proteins were different, underscoring the differences between the regulation of mRNA and proteins.

All gene expression data in which age was significantly affected, gene expression decreased over the course of the experiment. This effect was particularly pronounced in AvBDs. Avian beta defensins are important in innate immune responses and act directly against microorganisms, such as bacteria and fungi [38,58]. The AvBD genes showed the most profound changes in expression during this study. Only two AvBD genes (*AvBD8* and *10*) were affected by the interactions of feed and age, and *AvBD1* was found to be expressed significantly higher in NONFED birds; however, all were affected by the age of the birds. The work presented here is, to the best of our knowledge, the first comparison of AvBD gene expression between birds with immediate or delayed access to feed PH. At this point in time, the significance of the differences in the expression of *AvBD8* and *9* in FED and NONFED birds in unclear; however, these changes were observed between 24 and 72 h PH when differences in feed status occurred, suggesting that the innate immune function of chicks can be altered by access to feed. In the current study, the expression of AvBD genes began decreasing at time of hatch and from 72–144 h PH reached steady levels. The expression of *AvBD8* and *9* genes (which were affected by the interaction between age and feed status) as well as *AvBD10* were characterized by an initial increase in expression followed by a decrease. While these results are consistent with a previous study where AvBD genes expression were low by week 7 PH in the duodenal loop of chickens compared to their expression at hatch [59]; they are contrary to those reported by Terada et al. [60]. This study [60], measuring the gene expression of AvBDs in the ileum and ceca of e19 to D7 PH chicks, found that the expression of *AvBD8* and *10* were highest at D0, *AvBDs 1*, *2*, *4*, *6*, and *7* decreased following hatch, and *AvBDs 3*, *5*, and *12* showed no changes in expression. Lee et al. [59] reported that by week 7 PH, the expression of AvBD genes in the duodenal loop of chickens was low. A study measuring the gene expression of AvBDs in the ileum and ceca of e19 to D7 PH chicks, found that the expression of *AvBD8* and *10* were highest at D0, *AvBDs 1*, *2*, *4*, *6*, and *7* decreased following hatch, and *AvBDs 3*, *5*, and *12* showed no changes in expression [60]. In the current study, the expression of AvBD genes began decreasing at time of hatch and from 72–144 h PH reached steady levels. The expression of *AvBD8* and *9* genes (which were affected by the interaction between age and feed status) as well as *AvBD10* were characterized by an initial increase in expression followed by a decrease. When comparing existing studies, the results observed here do not conform to those observed in the ileum and ceca reported by Terada et al. [60]. These findings underscore the need to characterize the expression of the genes, proteins, and their function in a holistic manner in order to begin to understand the function of gut-associated immunity.

The final aim of this study was to investigate any correlation between the expression of cytokine and defensin genes and gut microbiota. For this study, the contents as well as mucosal scraping were collected from the ileum so that both adherent and non-adherent microbial populations could be tested since these populations vary and most likely have different effects in the gut. One of the obvious differences in the correlation study was that the expression of more genes had significant correlation with the presence of gut bacteria in NONFED compared to FED birds, particularly in the ileum scrapings (at family, genus, and species levels) and in ileum contents (family level). The correlation between the expression of AvBDs and specific bacterial populations in NONFED birds were clearly evident. For example, the correlation between the presence of the Lachnospiraceae family, “other bacteria”, and unclassified bacteria, the genus *Clostridium* and *Clostridium perfringens,* and the expression of many of the AvBDs was much more pronounced in NONFED birds. Avian beta defensins are expressed at hatch throughout the intestine, play important roles in the innate defense mechanism, and regulate the intestinal microbiota [61,62]. Considering these results, it is possible that in NONFED birds, the expression of AvBDs is regulated in response to changes in the microbiota. The genus *Clostridium* and, in particular, *Clostridium perfringens* have been linked to dysbiosis and incidences of necrotic enteritis (in conjunction with the presence of *Eimeria*) [63]. An attractive hypothesis would be that, in NONFED birds, dysbiosis caused by lack of access to feed induces the expression of AvBDs, which can directly mitigate bacteria. However, in the present study, only AvBD1, 8, and 9 were affected by access to feed. These findings underlie the complexity of the mechanisms that control gut health in growing broilers. Even though *Clostridium perfringens* can lead to necrotic enteritis, other members of the Clostridiaceae and Lachnospiraceae families have positive effects on the gut by producing short chain fatty acids by fermenting plant polysaccharides [64,65]; therefore, the correlation of their presence with the expression of AvBD genes in NONFED birds may be due to positive effects of these bacteria on the gut. Most of the significant correlations described here for AvBD genes were positive, meaning that higher gene expression was associated with greater presence of various microbiota. However, in the ileum contents the Enterococcaceae family was negatively correlated with gene expression in FED birds but positively correlated in NONFED birds. At the genus level, *Enterococcus* was negatively correlated with AvBD genes in both FED and NONFED birds. Again, the significance of this is not known; however, it shows differences between the interplay of the microbiota and immune function among birds with immediate access to feed versus delayed access to feed.

In addition to the correlation with AvBD genes, most genera were correlated with pro- and anti-inflammatory cytokines. For example, the genus *Enterococcus* was negatively correlated with anti-inflammatory (*IL-4* and *TGF-β*) and pro-inflammatory cytokines (*IL-6*), regardless of feeding status PH, suggesting that some *Enterococcus* may play an important role in modulating the immune system. The genus *Enterococcus* comprises of pathogenic and commensal bacteria [66], which are used as probiotics to reduce the negative effect of bacterial infection in chickens [67]. In contrast to *Enterococcus, Clostridium* was not correlated with any gene in the fed birds, but it showed a positive correlation with anti-inflammatory (*IL-4*) and pro-inflammatory cytokines (*IFN-β*) in NONFED birds. Although it is unclear why these two cytokines were positively correlated with *Clostridium* in NONFED birds, this positive correlation suggests that the access to feed plays an important role in the establishment of the intestinal microbiota and birds having early access to feed PH may benefit from healthy intestinal microbiota. Contrary to *Clostridium, Blautia* was negatively correlated with anti-inflammatory cytokines (*IL-4*) in FED and NONFED birds, suggesting the potential immune modulatory function of this genus. Another genus, *Streptococcus,* was positively correlated with the pro-inflammatory cytokine *IL-1β* only in NONFED birds. At the species level, *Streptococcus luteciae* showed positive correlation with *IL-1β* only in the scrapings of NONFED birds. Although some *Streptococcus* species are common inhabitants of the intestine, stressful conditions such as the lack of feed may negatively affect the intestinal mucosa in hatchlings [68] and lead to infection and the pro-inflammatory response of the immune system. It is important that future research be conducted to clarify the impact of feed delay on the relation between the intestinal microbiota and their immunomodulatory function.

## 5. Conclusions

The objective of the research presented here was to investigate the effect of withholding feed from newly hatched chicks on the expression of various cytokine and AvBDs genes in the ileum. Secondly, the study was extended to carry out a correlation analysis between gene expression and components of the ileal microbiota. The gene expression component of the study found that several transient significant interactions between the two main effects of age and treatment were present in the expression of several immune-related genes (*TGF-*β, *TLR4*, *IFN-γ*, *IL-1β*, *IL-4*, *IL-6*, and *AvBDs 8* and *9*). These effects were noted primarily at the end of feed delay and a day or two following feed delay. The remaining genes (*TLR2*, *IFN-β*, *IL-8*, *IL-18*, and *AvBDs 1–7* and *10*) showed a decrease in mRNA expression following hatch, with AvBDs showing a steep decrease in expression. The four proteins (IL-1β, IL-10, IL-8, and IL-18), whose expression was measured by ELISA, were only affected by bird age, suggesting that effects of feed delay on gene and protein expression may be different, underlining the importance of further development of chicken-specific protein reagents. In the correlation analysis between the gene expression and components of ileal microbiota, it was found that in NONFED birds there were more positive correlations than in FED birds, indicating that delayed access to feed affects the interplay between the immune response and intestinal microbiota. The increased number of significant correlations between immune-related genes and the genus *Clostridium*, *Enterococcus*, and *Clostridium perfringens* in FED and NONFED birds suggests a perturbation of the immune response and ileal microbiota in response to a lack of feed immediately PH. These results point out the complexity of the interplay between microbiota and the immune response and will hopefully help further explain the negative effects on production parameters in feed-delayed hatchlings.

## Figures and Tables

**Table 1:** Gene-specific primers used for the analysis of mRNA levels using quantitative real-time RT-PCR ^a^.

Gene ^b^	GenBank AccessionNo. ^c^	Forward Primer (5′→3′)	Reverse Primer (5′→3′)	Amplicon Size (bp)
*IL*-1β	NM_204524	GCATCAAGGGCTACAAGCTC	CAGGCGGTAGAAGATGAAGC	131
*IL-4*	NM_001007079	GAATGACATCCAGGGAGAGG	AACAATTGTGGAGGCTTTGC	112
*IL-6*	NM_204628	GGCATTCTCATTTCCTTCTAGG	CCGTAAGAAATGTAACAGGTGTTTT	135
*IL-8*	DQ393272	ATGTGAAGCTGACGCCAAG	GGCCATAAGTGCCTTTACGA	131
*IL-18*	NM_204608	TGAAATCTGGCAGTGGAATG	CAACCATTTTCCCATGCTCT	144
*TGF-β*	NM_001318450	CGACCTCGACACCGACTACT	CCACTTCCACTGCAGATCCT	135
*TLR2*	NM_204278	TCACAGGCAAAATCACGGTG	GATTTGGTTGGACTGCAGCA	116
*TLR4*	NM_001030693	TTCCTGCTGAAATCCCAAAC	TATGGATGTGGCACCTTGAA	132
*IFN-β*	NM_001024836	GTGCTTGTACCTGGGACCAT	GGATGAGGCTGTGAGAGGAG	107
*IFN-γ*	NM_205149	GCCGCACATCAAACACATATCT	TGAGACTGGCTCCTTTTCCTT	207
*AvBD1*	NM_204993	TGTGCATTTCTGAAGTGCCC	TTGGGATGTCTGGCTCTTCA	104
*AvBD2*	NM_204992	GTTCCGTTCCTGCTGCAAAT	TGAGAGGGGTCTTCTTGCTG	133
*AvBD3*	NM_204650	GATTCTGTCGTGTTGGGAGC	TCCTCACAGAATTCAGGGCA	114
*AvBD5*	NM_001001608	ATTACCCCAGGACTGTGAGC	ACGTGAAGGGACATCAGAGG	147
*AvBD6*	NM_001001193	GCCCTACTTTTCCAGCCCTA	CCTGTTCCTCACACAGCAAG	133
*AvBD7*	NM_001001194	CTCTTGCTGTGCAAGGGGAT	GGAGTGCCAGAGAAGCCATT	91
*AvBD8*	NM_001001781	ATGCTCCAAGGATCACTGCT	CTGCTTAGCTGGTCTGAGGT	122
*AvBD9*	NM_001001611	GACGCTGACACCTTAGCATG	CCCATTTGCAGCATTTCAGC	118
*AvBD10*	NM_001001609	CACTTTTCCCTGACACCGTG	AAAGCCTTTCCTTACTGCGC	148

^a^ All primers used for expression analysis were designed using the primer3 program (http://bioinfo.ut.ee/primer3-0.4.0/primer3/) [43]. ^b^ Abbreviations of the gene names are defined in text. ^c^ Reference chicken gene sequences that contain the corresponding PCR products list.

## Data Availability

The 16S rRNA gene sequences determined in this study were deposited in the NCBI Sequence Read Archive (SRA) database (SRA accession #PRJNA779402). The data that support the findings of this study are available from the corresponding author, K.B.M., upon reasonable request.
